# Effects of Undernutrition and Predictors on the Survival Status of HIV-Positive Children after Started Antiretroviral Therapy (ART) in Northwest Ethiopia

**DOI:** 10.1155/2022/1046220

**Published:** 2022-02-17

**Authors:** Mulugeta Molla, Fassikaw Kebede, Tsehay Kebede, Assefa Haile

**Affiliations:** ^1^Pharmacology and Toxicology Unit, Department of Pharmacy, College of Health Sciences, Debre Tabor University, Debre Tabor, Ethiopia; ^2^Department of Epidemiology and Biostatistics, School of Public Health, College of Health Science, Woldia University, Woldia, Ethiopia; ^3^Department of Geography and Environmental Study, Faculty of Social Science, Bahir Dar University, Bahir Dar, Ethiopia; ^4^Department of Nursing and Midwifery, Pawe Health Science College, Metekel Zone, Pawe Woreda North West, Ethiopia

## Abstract

Malnutrition and human immunodeficiency virus/acquired immunodeficiency syndrome have complex and multidirectional relationships. Ethiopia is one of the countries hardest hit by the HIV epidemic as well as malnutrition. This study was aimed at assessing the effects of undernutrition on the survival status of HIV-positive children who received HIV/AIDS care in Northwest Ethiopia. *Materials and Methods*. A facility-based retrospective follow-up was conducted from January 1, 2009, to December 31, 2020. The data was entered into EpiData version 4.2.0. Then, the entered data was exported to STATA 14 software for further analysis, and the Kaplan-Meier survival curve was used to estimate survival time after the initiation of ART. The Bivariable and multivariable Cox regression analyses were conducted to identify predictors of mortality associated with undernutrition. *Results*. The mean (±SD) age of participant children was found 118.4 (±38.24) months. The overall mortality rate in this study was determined as 5.4 per 100 child-years (95% CI: 3.6, 5.8). Children with CD4 cell counts below the threshold [AHR = 1.6; 95% CI (1.19, 7.85)], advanced WHO clinical stages (III and IV) HIV [AHR = 4.5; 95% CI (2.80, 8.40)], and being severe stunting at the beginning [AHR = 2.9; 95% CI (1.80, 6.40)] were significantly associated with mortality of HIV-positive children. *Conclusion*. The findings of the current study indicated that HIV-positive children on ART had a high rate of mortality. Baseline undernutrition has the mortality of children who had CD4 counts below a threshold, advanced WHO HIV clinical staging (III and IV), and being severe stunting (HAZ ≤ −3 Z score) which were found to be independent predictors for mortality of undernourished HIV.

## 1. Introduction

Malnutrition and human immunodeficiency virus (HIV)/acquired immunodeficiency syndrome (AIDS) have complex and multidirectional relationships that cause progressive immune system damage [1]. Both are frequently intertwined and have a synergistic effect [2]. Malnutrition increases viral replication and accelerates the progression of HIV disease by decreasing CD4 T cells, suppressing delayed hypersensitivity, and altering *β*-cell responses [3, 4]. For this reason, malnutrition has a poor prognosis for clinical outcomes [5]. However, HIV/AIDS accelerates the progression of immune impairment and the occurrence of opportunistic infections [6] by increasing the risk of appetite loss, hastening the decline of CD4 T cell concentration, and causing markers of microbial translocation (16sDNA), intestinal damage (iFABP), monocyte activation, and increased proteolysis in HIV-positive children [6–10]. Children with HIV/AIDS have a reduced appetite and ability to consume food, as well as a higher incidence of diarrhea, resulting in malabsorption and nutrient a loss, which makes malnutrition a common phenomenon [11, 12]. In 2018, an estimated 1.7 million children (aged <15 years) were living with HIV worldwide [13, 14]. In the same year, in Ethiopia, 44229 children were diagnosed with HIV and 2055 died related to AIDS [9, 15]. Undernutrition in all its forms remains a global public health burden that accounts for 45% of all deaths for people living with HIV [2, 16]. Children are more vulnerable to undernutrition than adults with HIV; hence, rapid viral replication and a higher rate of CD4 cell destruction are inevitable due to immature immunity [17]. Evidence suggests that even relatively small losses in weight (5%) are associated with a decrease in the survival rate of HIV-positive children [18]. Epidemiologically, the incidence of stunting is declining too slowly, while wasting still has a great impact on too many young children worldwide [19]. In 2017, 13.8 million children were wasted, of whom 4 million were severely wasted [13, 20]. Most of all were in sub-Saharan countries [21]. Each year, over a million children die and develop severe acute malnutrition (SAM), and it presents mainly in marasmus, kwashiorkor, or marasmus kwashiorkor [22, 23]. Similarly, in comparison to kwashiorkor, wasting was more commonly reported [8, 24], as was early bone growth failure and an increased risk of death in HIV-positive children [1, 25].

Several studies investigated whether lower CD4 cell count, age, wealth index, adherence, food insecurity, and social support were risk factors for malnourished HIV-positive children's mortality [1, 2, 15, 26–28]. Ethiopia is one of the countries hardest hit by the HIV epidemic as well as malnutrition [1]; the prevalence of undernutrition among HIV/AIDS children ranges from 12.3 to 46.8% [19, 29] and is blamed for 57% of deaths [6, 13]. However, evidence of the effect of undernutrition on the survival status of HIV-positive children who are <15 years of age remains sparse and inconclusive [11, 29]. This study aims to assess the effects of undernutrition and its predictors on the survival of HIV-positive children who received antiretroviral therapy (ART) in selected public health facilities in Northwest Ethiopia.

## 2. Materials and Methods

### 2.1. Study Design and Setting

A facility-based retrospective follow-up study was employed among 721 HIV-positive children who started HIV/AIDS care in two hospitals and two health centers since January 1, 2009, to December 31, 2020, which are representative of the Benishangul-Gumuz region. All these health facilities provide health care services for all Oromia, Amhara, and Benishangul regions [30]. Following HIV/AIDS care initiated in the regions between January 1, 2009, and December 31, 2020, there were 2968 HIV/AIDS care started populations. More than 737 of these populations were children living with HIV/AIDS, and the data were drawn from two hospitals and two health centers (Assosa Hospital *N* = 359 and Pawe Hospital *N* = 315; Felege Selam Health Center *N* = 21 and Gilgel Beles Health Center *N* = 37) which were included for the final data analysis.

### 2.2. Populations

The records of all HIV-infected children, whoever started ART in four health institutions, were the source of the population. The records of all HIV-infected children receiving ART between January 1, 2009, and December 31, 2020, were considered as the study population. All HIV-infected children who had at least one month of ART follow-up from January 1, 2009, to December 31, 2020, were included.

### 2.3. Sample Size Determination and Sampling Techniques

The sample size was calculated by using the formula [31] for survival analysis by considering two-sided significance level (*α* = 5%), Z*α*/2 = Z value at 95%confidence interval = 1.96, power (Z*β*) = 80%, and *P* = %cumulative occurrence of death rate, 1.65 HR.

The final sample size (*N*) = *E* = (*Z* *α*_/2_ ± *Zβ*)^2^ = (*Zα*/2 + *Zβ*),

(1) $$\begin{array}{c} P\left(E\right)\;\theta^{2}p\left(1-p\right),\;p\left(1-\text{p}\right){\left(\text{lnHR}\right)}^{2},\\ \theta =\ln\left(\text{HR}\right),\\ \text{HR}={\text{e}}^{\theta }, \end{array}$$

where *α* = 0.05, *β* = 0.2, HR = hazard ratio, *N* = sample size, *E* = number of events, *P* (*E*) = probability of event, and *P* = cumulative occurrence of treatment failure, used as reference for sample size calculation [32]. The final sample size was determined as 512.5 after adding a 15% contingency for incompleteness. From January 1, 2009, to December 31, 2020, in four health institution there were totaly 738 recoded chartes of HIV infected children; while with outany sampling procedure we included all 738 files, since it is manageable by resource and human poweres for stastical analysis and increases stastical inferen.

### 2.4. Outcome Ascertainment

The outcome variable for this study is the death of HIV-positive children after starting HIV/AIDS care. Death is defined as when the child has approved records of death in his/her treatment follow-up medical records after initiating HIV/AIDS care from January 1, 2009, to December 31, 2020, where it is not due to accidental unrelated causes. Those records or ART clients who did not develop the outcome (death) at the end of the study are called censored cases.

### 2.5. Independent Variables

The independent variables were sociodemographic, clinical, and medication-related variables, as well as nutritional. Sociodemographic variables included age and sex of children, caregivers' age and residence, marital status of the caregiver, family size, caregivers' relationship to the child, and religion. Baseline clinical and hematologic variables included the World Health Organization (WHO) clinical staging, CD4 count, hemoglobin, and opportunistic infections. ART-related variables included regimen, duration of ART, regimen change, treatment failure, isoniazid preventive therapy (IPT), cotrimoxazole preventive therapy (CPT), and adherence status of ART. Nutritional status included weight for age (W/age), height for age (H/age), and weight for height (W/H).

### 2.6. Operational Definitions

#### 2.6.1. Severe Acute Malnutrition (SAM)

According to the WHO, children under the age of 59 months who have a weight-for-height ratio of ≤ -3 Z score, a mid-upper arm circumference of <15 cm, bilateral edema, and a failed appetite test should be admitted for inpatient care [33].

#### 2.6.2. Undernutrition

Undernutrition was defined as a child having one of the following descriptions: WFA Z score < −2 or HFA Z score < −2 or WFH Z score < −2 SD [3, 13].

#### 2.6.3. Nutritional Status

A well-nourished child had a W/age Z score > −2, H/age Z score > −2, or W/H Z score > −2 SD and/or mid-upper arm measurement (MUAC > 11.5 cm) with no pathological or physiological grading edema [6].

#### 2.6.4. CD4 Count

CD4 below the threshold level was classified based on the age of the child (i.e., infants CD4 < 1500/mm^3^, 12–35 months <750/mm^3^, 36–59 months <350/mm^3^, and ≥5 years <200/mm^3^) [23].

#### 2.6.5. ART Adherence

ART adherence for pediatrics is classified based on the percentage of drug dosage calculated from the total monthly doses of ART drugs: good > 95%, fair = 85 − 94%, and poor < 85% [34].

### 2.7. Data Collection Instruments

Before the data collection procedure was deployed for children, the standard anthropometric measurements were taken from all subjects at recruitment and at follow-up after being discharged from the clinic. MUAC was measured using a color-coded MUAC tape on the left arm. Data abstraction tools (checklists) were prepared using Ethiopia's Federal Ministry of Health Pediatrics ART follow-up and medical history sheet combination [35]. Six diploma nurses and four BSc public health officers were recruited for data collection and supervision. One day of training was given for data collection and supervision for all facility data collectors.

### 2.8. Data Collection Procedures and Quality Assurance

To assure the quality of the data, data collectors and supervisors were trained about how and what information they should collect from the medical records for one day. The checklist was pretested on 5% of randomly chosen charts that were not included in the actual study. After the pretest, the necessary modifications to the data collection tool were made. Strict follow-up and supervision were carried out during data collection by the principal investigator, and feedback was given daily. Individual records with incomplete data during data collection were excluded. The collected data was first checked and cleaned for completeness.

### 2.9. Data Processing and Analysis

Following data collection and quality assurance, the questionnaire was coded and the data entered into EpiData version 4.2.0. Then, the entered data was exported to STATA 14 software and used for analysis. The proportional hazard assumption was checked for each variable, and no variable was found with a Schoenfeld residual test <0.05. The Kaplan-Meier survival curve was used to estimate the survival time after the initiation of ART, and log-rank tests were used to compare the survival curves. Accordingly, the final Cox regression model was fitted based on final step selected variables after model assumption, and three variables were associated with undernutrition-related mortality of HIV-positive children with 95% CI at *P* < 0.05 and claimed as the predictor for death [36].

## 3. Results

### 3.1. Sociodemographic Characteristics of Both HIV-Positive Children and Caregivers

Out of 732 records, 721 were included, resulting in a response rate of 98.50%. About half of the 384 (53.26%) were females. The largest percentage of children was categorized under the age group of ≥121 months, which accounted for 389 (53.96%) of the total subjects, with a mean (±SD) age of 118.4 ± 38.24 months. The majority 510 (70.74%) of children living with HIV were in urban residences. The majority, 498 (69.07%), of caregivers were married, while their mean ± SD was 58.1 ± 18.6 years. Regarding the family size of the caregivers, 227 (31.48%), 462 (64.08%), and 32 (4.44%) of the HIV-positive children had less than two, three to five, and more than five family sizes, respectively. More than two-thirds (76.28%) of the children's caregivers were HIV-positive. Furthermore, 381 (52.84%), 337 (46.74%), and 381 (52.84%) of child caregivers were Orthodox religious believers and merchants by occupation, and both parents were alive, respectively (Table 1).

### 3.2. Clinical Characteristics of Study Participants

One hundred twenty-eight (17.75%) of the 721 children were diagnosed with SAM and hospitalized for inpatient therapy. More than one-third of the cases (35.78%) had at least one form of opportunistic infection before starting ART. The majority of children, 293 (40.64%), were on AZT-3TC-NVP of the ART regimen. In terms of adherence to ART at the time of the most recent ART visit, 356 (49.38%) of the participants had good adherence, whereas 188 (26.07%) of them had poor adherence. During enrollment in chronic HIV care, 237 (32.87%) and 202 (28.02%) of children on ART were on WHO stages I and II, respectively, while the remaining 282 (39.11%) cases were on stages III and IV. A majority, 451 (62.55%) and 419 (58.11%), received isoniazid preventive therapy and cotrimoxazole preventive therapy, respectively. More than a third of the children, 308 (42.72%), had CD4 counts below the threshold for severe immunodeficiency. Two hundred ninety-five (40.92%) cases had 37–72 months of follow-up (Table 2).

### 3.3. Nutritional Status of the Study Participants

At baseline, approximately one-fifth of the participants (19.97%) were moderately underweight, 21.36% were moderately stunted, and 16.50% were moderately wasted. Furthermore, the proportions of presenting child malnutrition cases were classified as severe underweight (11.93%), severe stunting (19.28%), and severe wasting (9.85%) (Table 3).

### 3.4. Survival Status of HIV-Positive Children

The study participants were followed for 20116.845 person per month (PMOS) of risk observation within a median follow-up period of 23.56 months (IQR = ±13.3 months). At this instant, 30 (4.2%) cases had died, with a median survival rate of 0.94 (95% CI: 0.91, 0.96). At the end of follow-up, 394 (54.62%) were currently on follow-up, whereas the remaining 212 (29.40%), 14 (1.94%), and 90 (12.48%) were gone up on adult cohort, abscond, and decease, respectively. The mean survival time of the entire follow-up was 28.49 months (95% CI: 26.7, 59.5). The cumulative probabilities of survival rate at four, eight, 16, 24, 48, and 72 months after the initiation of ART were 0.99, 0.98, 0.97, 0.94, 0.71, and 0.49, respectively. The overall mortality rate in this study was determined as 5.4 per 100 child-years (95% CI: 3.6, 5.8).

### 3.5. Log-Rank Estimate of Mortality and Comparison of Death Hazard

**Figure 1:**
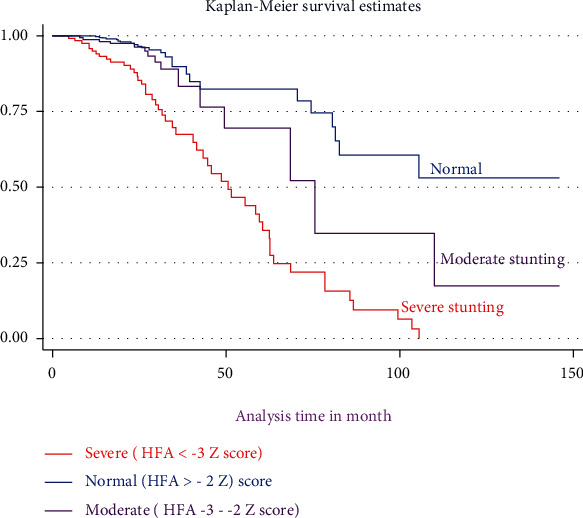
The Kaplan-Meier survival curves comparing the survival times of HIV-positive children on ART with different categories of stunting among selected public health facilities in Northwest Ethiopia from January 1, 2009, to December 30, 2020.

**Figure 2:**
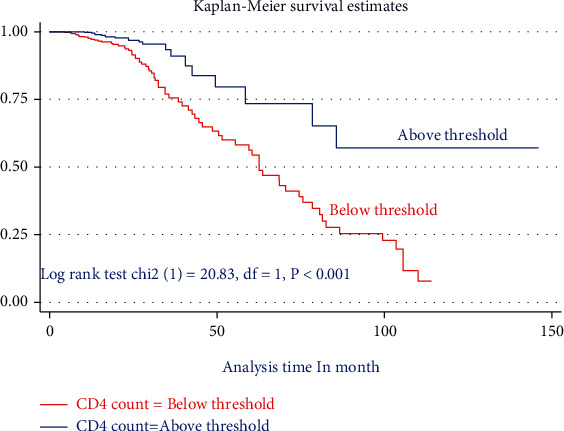
The Kaplan-Meier failure estimate curves to compare the hazard of death for HIV-positive children on ART with different categories of CD4 count in the selected public health facilities in Northwest Ethiopia, January 1, 2009–December 30, 2020.

**Figure 3:**
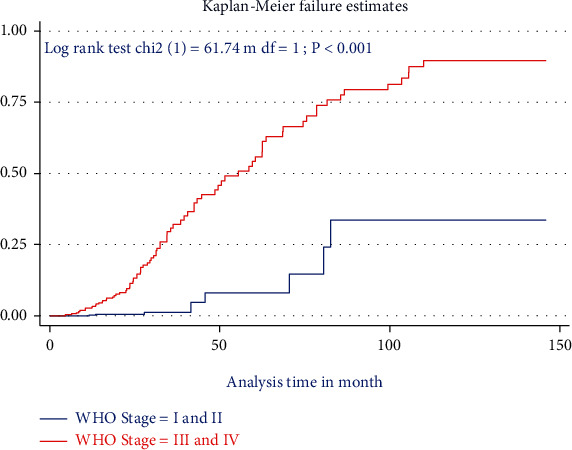
The Kaplan-Meier failure estimate curves to compare hazard of death for HIV-positive children on ART with different categories of WHO clinical stages in the selected public health facilities in Northwest Ethiopia, January 1, 2009–December 30, 2020.

The Kaplan-Meier survival curve together with the log-rank test shows differences in the hazards of death or undernutrition in HIV-positive children on different covariates. Being HIV-infected children at baseline stunting (*HAZ* ≤ −3 Z score), being on WHO clinical stage (III and IV), and having CD4 count below threshold had survival differences between undernourished and counter peers do have (Figures 1–3).

### 3.6. Bivariable and Multivariable Cox Regression Analysis

As shown in Table 4 in the multivariable Cox regression analysis, after adjustment and controlling of certain confounding in the final model, three variables were found significantly associated with mortality of undernourished children. HIV positive children with CD4 cell counts below the threshold were 1.6 times at higher risk of death as compared to cohort members above the threshold [AHR =1.6; 95% CI, (1.19, 7.85)]. In the current study, WHO HIV clinical stages were found to be another predictor of mortality. Children with advanced WHO HIV clinical stages (III and IV) had a 4.5 times higher risk of death as compared to those with WHO HIV clinical stages (I and II) [AHR = 4.5; 95% CI (2.80, 8.40)]. The presence of severe stunting at the beginning of ART was associated with a 2.9 times higher risk of death than nonstunting cases [AHR = 2.9; 95% CI (1.80, 6.40)] (Table 4).

## 4. Discussion

In this retrospective cohort study, the effects of undernutrition and its predictors for survival status among HIV-positive children on ART were determined. At the end of chronic successive follow-up, about 90 (12.48%) patients were deceased, and 14 (1.94%) patients were absconded. The review data on the national level revealed a mortality rate of 5 to 8% at 6 months and 24 months after ART started [11, 23]. The overall mortality rate of this study was 5.4 per 100 child-years (95% CI: 3.6, 5.8), which is consistent with findings from the Felege Hiwot Comprehensive Specialized Hospital, Northwest Ethiopia (four deaths per 100 child-years) [37] and Northwest Ethiopia 4.4 per 100 child-years (95% CI: 3.2, 6.0) [13]. However, the mortality rate reported in this study is higher than studies reported from Mekelle Hospital, Northern Ethiopia (1.4 deaths per 100 child-years) [38], Wolaita Zone health facilities, Southern Ethiopia (2.1 deaths per 100 child-years) [39], Northwest Ethiopia (3.2 deaths per 100 child-years [29], the Asia-Pacific region (1.9 deaths per 100 child-years) [40], Zimbabwe (2.9 deaths per 100 child-years) [41], and Congo (3.4 deaths per 100 child-years) [42]. Conversely, the mortality rate found in this study is much lower than that of Debre Tabor General Hospital and Dessie Referral Hospital (6.3 deaths per 100 child-years) [38], Addis Ababa (8.8 deaths per 100 child-years) [13], Southwest Ethiopia (11.2 deaths per 1000 person-years) [11], and Kenya (8.4 deaths per 100 child-years) [43]. Explanations for variation in the incidence of mortality rate might be due to the difference in health care awareness of the community, sample size, study settings, study period, and/or characteristics of study participants. The present study explored predictors of mortality among study participants. In this study, children who had CD4 counts below the threshold showed a higher risk of death than their counterparts. This finding is consistent with other previous studies conducted in Ethiopia [11, 18, 19, 29, 37, 44], India [45], Congo [42], Tanzania [46], Bangladesh [47], and Malaysia [48], which all indicate that low CD4 count was an independent predictor of mortality. Serious and life-threatening opportunistic infections, such as central nervous system toxoplasmosis and cryptococcal meningitis, are more common in children with severe immunodeficiency. This hastened a sharp reduction in CD4 count; this has declined the survival probability of children since care started. Advanced WHO HIV clinical staging (III and IV) was another strong predictor of mortality. Children with advanced WHO HIV clinical stage (III and IV) at the time of ART initiation have a higher risk of death as compared to their counterparts with mild status (i.e., WHO HIV clinical disease stage (I and II)). Similar results were reported from previous studies conducted in Ethiopia [8, 37, 49, 50], two rural hospitals in South Africa [5], Tanzania [38], Zimbabwe [41], Kenya [43], Eastern India [45], and Malawi [51], which all indicated that advanced WHO HIV clinical disease stages were a predictor of mortality. For those living with HIV, as WHO HIV clinical staging becomes more advanced, the risk of developing and recurrence of opportunistic infection also increases, which might be associated with the cause of death.

In this study, severe stunting was also an independent predictor of mortality among HIV-positive children on ART. Children who were severely stunted before ART initiation have a higher risk of death as compared to those who were not stunted. This is consistent with findings in Northwest Ethiopia [13], Burkina Faso [4], and Tanzania [22]. Reasonably, malnutrition is a common complication of HIV infection. Severe stunting is associated with a weakened immune system and complicates the treatment of diseases by affecting intestinal absorption of drugs and the ability to absorb various nutrients, besides causing dysregulated lipid metabolism and increased proteolysis in the body [1, 8, 46].

## 5. Limitations of the Study

There were inconsistencies in determining causes of death in this study, particularly for individuals who died at home. Some of those who died without being notified may have been counted as “lost to follow-up.” In addition, important determinants of death, such as viral load and nutritional deficiencies, were also not taken into account.

## 6. Conclusion

The findings of the current study indicated that HIV-positive children on ART had a high rate of mortality. In this study, nutritional status was found to have a significant effect on the survival of HIV-positive children on ART. Baseline CD4 counts of less than the threshold, advanced WHO HIV clinical disease staging (III and IV), and severe stunting were found to be independent predictors of mortality in HIV children on ART. This calls for the government to give due attention to strengthening HIV/AIDS treatment and care modalities and ensure that the relevant nutritional support for children at risk is provided appropriately.

## Figures and Tables

**Table 1:** Sociodemographic characteristics of HIV-positive children attending ART care in selected public health facilities in Northwest Ethiopia, January 1, 2009–December 30, 2020.

Variables	Categories	Frequency	Percent
Sex	Female	384	53.26
Age of children	≥5 years	78	10.81
	6–10	254	35.23
	≥11 years	389	53.96
HIV disclosure status of children	Disclosed	158	21.91
Age of caregivers	≤45 years	244	33.84
Resident	Urban	510	70.74
Marital status of the caregiver	Single	115	15.95
	Married	498	69.07
	Divorced	82	11.37
	Widowed	26	3.61
Family size of caregivers	≤2	227	31.48
	3–5	462	64.08
	≥6	32	4.44
HIV status of caregivers	Positive	550	76.28
	Negative	91	12.62
	Unknown	80	11.10
Religion of caregivers	Orthodox	381	52.84
	Muslim	152	21.08
	Protestant	139	19.28
	Catholics	49	6.80
Occupational status of caregivers	Farmer	99	13.73
	Merchant	337	46.74
	Employer	124	17.20
	Laborer worker	161	22.33
Parental status of children	Both alive	381	52.84
	Paternal orphan	135	18.72
	Maternal orphan	108	14.99
	Both orphaned	97	13.45
Income class of caregiver	First quintile	288	39.94
	Second quintile	123	17.06
	Third quintile	85	11.79
	Fourth quintile	119	16.51
	Fifth quintile	106	14.70

**Table 2:** Clinical and immunological profiles of HIV-positive children who received ART care in selected public health facilities in Northwest Ethiopia, January 1, 2009–December 30, 2020.

Variables	Categories	Number	Frequency
Dietary counseling during follow-up	Yes	465	64.49
Admission history of SAM	Yes	128	17.75
Opportunistic infections at baseline	Yes	258	35.78
ART regimen types	D4T-3TC-NVP	48	6.66
	D4T-3TC-EFV	26	3.61
	AZT-3TC-NVP	293	40.64
	AZT-3TC-EFV	165	22.88
	TDF-3TC-EFV	104	14.42
	AZT-3TC-LPV/R	36	4.99
	ABC-3TC-NVP	25	3.47
	ABC-3TC-EFV	24	3.33
ART regimen change	Yes	211	29.26
Functional status (age ≤ 5 years)	Appropriate	68	71.58
	Delay	15	15.79
	Regression	12	12.63
Developmental history (age > 5 years)	Working	488	77.96
	Ambulatory	87	13.89
	Bedridden	51	8.15
Adherence	Good	356	49.38
	Fair	177	24.55
	Poor	188	26.07
WHO clinical stage	I	237	32.87
	II	202	28.02
	III	170	23.58
	IV	112	15.53
Isoniazid preventive therapy	Yes	451	62.55
Cotrimoxazole preventive therapy	Yes	419	58.11
CD4 count	Below the threshold	308	42.72
Hemoglobin level	≤10 g/dl	229	31.76
ART eligibility criteria	Immunologic	81	11.23
	Clinical stage	96	13.31
	WHO clinical stage	79	10.96
	CD4 threshold	199	27.60
	Examine and treat	266	36.90
Type of opportunistic infection	Bacterial pneumonia	79	30.62
	Diarrhea	74	28.68
	Meningitis	9	3.49
	Pneumocystis pneumonia	6	2.32
	Skin dermatitis	7	2.71
	Kaposi's sarcoma	5	1.94
	Acute/chronic otitis media	9	3.49
	Others	3	1.16
	Tuberculosis	66	25.59
Duration of ART	≤36 months	223	30.93
	37–72 months	295	40.92
	>72 months	203	28.15
Maternal PMTC follow-up history	Yes	487	67.55
MUAC	≤11.5 cm	270	37.45
Tuberculosis treatment history	Yes	66	9.15
Survival status of children	Died	87	12.07

**Table 3:** Nutritional status of HIV-positive children who received ART care in selected public health facilities of Northwest Ethiopia 2009-2020.

Variables	Categories	Frequency	Percent
Underweight (W/age)	Normal	491	68.10
	Moderate underweight (WAZ ≤ −2)	144	19.97
	Severe underweight (WAZ ≤ −3)	86	11.93
Stunting (H/age)	Normal	428	59.36
	Moderate stunting (HAZ ≤ −2 Z score)	154	21.36
	Severe stunting (HAZ ≤ −3 Z score)	139	19.28
Wasting (W/H)	Normal	531	73.65
	Moderate wasting (WHZ or BAZ ≤ −2)	119	16.50
	Severe wasting (WHZ or BAZ ≤ −3)	71	9.85

**Table 4:** Bivariable and multivariate Cox regression for predictors of mortality of undernourished HIV-positive children among selected health facility of Northwest Ethiopia 2009-2020.

Variables	Categories	Survival status		CHR (95% CI)	AHR (95% CI)
		Died	Censored		
Sex	Male	35	302	1	1
	Female	56	328	1.3 (0.88, 2.07)	1.7 (0.70, 1.70)
Age	≤60 months	29	49	2.8 (1.70, 4.60)	1.7 (0.79, 3.20)
	61–120 months	23	231	1.2 (0.79, 2.11)	2.5 (0.93, 4.50)
	≥121 months	38	351	1	1
Residence	Urban	61	449	1.16 (0.73, 1.87)	1.12 (0.7, 1.85)
	Rural	29	182	1	1
CD4 count	Below threshold	70	238	3.12 (1.90, 7.20)	1.6 (1.19, 7.85)^∗^
	Above threshold	20	393	1	1
WHO stage	Stages I and II	8	435	1	1
	Stages III and IV	82	196	10.9 (5.20, 22.70)	4.5 (2.80, 8.40)^∗^
Adherence	Optimal adherence	21	489	1	1
	Suboptimal adherence	69	142	6.3 (3.80, 10.30)	1.8 (0.60, 3.60)
Dietary counseling	Yes	44	421	1	1
	No	46	210	1.8 (1.50, 2.50)	1.9 (0.50, 1.50)
Disclosure status	Yes	54	104	3.3 (2.30, 5.10)	1.7 (0.59, 2.8)
	No	36	527	1	1
Duration of follow-up	≤36 months	6	217	1	1
	37–72 months	19	276	2.1 (0.90, 5.10)	2.1 (0.70, 5.70)
	>72 months	65	138	7.1 (3.10, 16.30)	2.6 (0.80, 3.60)
Wasting	Normal	70	461	1	1
	Moderate (WHZ or BAZ < −2)	18	101	9.6 (1.28, 12.6)	0.8 (0.18, 4.30)
	Severe (WHZ or BAZ < −3)	2	69	7.8 (1.80, 16.30)	1.07 (0.50, 1.90)
Underweight	Normal	34	457	1	1
	Moderate (WAZ < −2)	18	126	1.6 (0.91, 2.91)	0.6 (0.30, 1.17)
	Severe (WAZ < −3)	38	48	2.9 (1.80, 4.82)	1.2 (0.60, 2.1)
Stunting	Normal	18	410	1	1
	Moderate (HAZ < −2)	29	125	5.6 (2.50, 8.40)	1.6 (0.80, 3.20)
	Severe (HAZ < −3)	43	96	4.6 (3.20, 9.90)	2.9 (1.80, 6.40)^∗^
MUAC	≤11.5 cm	56	214	4.5 (2.10, 6.10)	1.5 (0.60, 4.20)
	>11.5 cm	34	417	1	1
CPT	Yes	17	402	1	1
	No	73	229	2.5 (1.60, 7.60)	1.2 (0.43, 4.50)
SAM admission history	Yes	37	91	2.8 (1.80, 4.30)	1.7 (0.60, 2.90)
	No	53	540	1	1

CHR = crude hazard ratio; AHR = adjusted hazard ratio; CI = confidence interval; 1 = reference category. ^∗^Significant predictors variables on final the multivariable models at *P* < 0.05.

## Data Availability

All relevant data were within the corresponding author upon reasonable request.

## Abbreviations

ART: Antiretroviral therapy
AIDS: Acquired immunodeficiency syndrome
HAZ: Height for age Z score
HIV: Human immunodeficiency virus
SAM: Severe acute malnutrition
WAZ: Weight for age Z score
WHO: World Health Organization
WHZ: Weight for height Z score.
